# Production of Activated Biochar Derived from Residual Biomass for Adsorption of Volatile Organic Compounds

**DOI:** 10.3390/ma16010389

**Published:** 2022-12-31

**Authors:** Elena David

**Affiliations:** National Research Institute for Cryogenic & Isotopic Technologies, Street Uzinei no. 4, P.O. Râureni, P.O. Box 7, 240050 Râmnicu Vâlcea, Romania; elena.david@icsi.ro or elenadavid2004@yahoo.com

**Keywords:** activated biochar, residual biomass, carbonization, chemical activation, VOC adsorption, environment application

## Abstract

Volatile organic compounds (VOCs) released in air represent a major potential for environmental pollution. Capture methods based on activated biochar have attracted attention because of their low cost and for the high removal capacity of the material due to its physical and chemical properties. In this paper, activated biochars were developed and their adsorption performance for VOC capture was evaluated. In the first step, biochars derived from rapeseed cake (RSC) and walnut shells (WSC) were obtained through a carbonization process and then were activated using basic/acid agents (KOH/H_2_SO_4_) to increase their performance as adsorbents. Acetone and toluene were used as the VOC templates. The adsorption capacities of toluene and acetone for non-activated biochars were reduced (26.65 mg/g), while that of activated biochars increased quite significantly, up to 166.72 mg/g, and the biochars activated with H_2_SO_4_ presented a higher adsorption capacity of VOCs than the biochars activated with KOH. The higher adsorption capacity of biochars activated with H_2_SO_4_ can be attributed to their large surface area, and also to their larger pore volume. This activated biochar adsorbent could be used with good results to equip air purification filters to capture and remove VOCs.

## 1. Introduction

The large-scale use of fossil fuels, as well as industrial activities, produce a variety of atmospheric pollutants that are harmful to both the environment and human health. Among them, volatile organic compounds (VOCs) are emitted in large quantities and come mainly from industrial production [1,2]. The discharge of volatile organic compounds into the atmosphere affects the quality of the environment and human health, because most of them are carcinogenic toxic compounds and produce by even more dangerous products by degradation, such as organic aerosols, that lead to the appearance of smog and the depletion of the ozone layer [3,4,5]. Therefore, it is necessary to develop efficient and ecological systems for the elimination of VOCs.

There are some methods for removing air pollutants, such as the separation method based on membranes [6], a method based on the adsorption process [7]. Additionally, an adsorption-based method is usually used to remove air pollutants [8]. Other methods based on the use of chemical compounds and catalysts increase the costs and can lead to supplemental pollution and equipment damaged by corrosion. Among the VOC removal methods, adsorption is considered one of the most suitable methods to remove VOCs, mainly because it is low-cost and high-efficiency [9]. The adsorption method has > 90% efficiency, moderate energy consumption and is easy to apply [10,11]. Air pollutants, such as CO_2_, H_2_S, NH_3_ or VOCs, can be removed by adsorption on porous materials with a high specific surface area [12,13].

The main problem is finding the optimal adsorbent that is the key factor for the commercial application of the adsorption technology. Carbon materials, in spite of some disadvantages such as hygroscopicity and pore blocking, are considered to have the most potential as a low-cost, high-efficiency, and good-stability adsorbent for VOC removal [14]. On the other hand, their large-scale use is still limited because of their high cost of production [15,16]. As a result, it is necessary to develop low-cost adsorbents which can efficiently retain pollutants from the air and thus reduce the risk to the environment and human health. In this context, activated biochar is considered a suitable material for such applications. Biochar is a porous carbonaceous material obtained by the conversion of biomass under an atmosphere with low oxygen content, or even in an inert atmosphere [17,18]. Due to its properties such as low cost [16], high adsorption capacity, and the activity of functional groups on the surface [19,20], biochar can be used in systems for removing air pollutants, such as VOCs. There are various methods to prepare and modify biochar for its applications in the removal of VOCs from air. The raw material frequently used for the production of biochar includes biomass, agricultural waste, sludge, manure, etc. [21,22], and the preparation methods typically used are pyrolysis, carbonization and hydrothermal carbonization or gasification [23,24]. The differences that exist between the raw materials used, as well as between the preparation methods, influence the physicochemical properties and implicitly the adsorption capacity of the biochar.

The gasification method consists of a partial oxidation and then combustion of raw materials in the presence of air or steam at a temperature in the range of 600 to 1200 °C; the main resulting product is gas and the biochar yield is small [25,26]. Hydrothermal carbonization refers to a method of processing of biomass at a temperature of 180–320 °C, and under a certain pressure, which was previously immersed in water. The biochar obtained by this method usually has high surface functionalized groups, high moisture content and acidity [27,28].

Another method of producing biochar is pyrolysis, which consists of a process of thermal decomposition of the raw material. The process takes place in an inert atmosphere or in an environment with a low concentration of oxygen [28]. There are two types of pyrolysis: slow and fast pyrolysis. Slow pyrolysis is used to produce a high yield of biochar, while fast pyrolysis produces a high yield of bio-oil and gas [29]. The slow pyrolysis process utilizes a slow heating rate, the residence time is long and the temperature is in a large range [30,31]. Based on pyrolysis temperature, slow pyrolysis is divided into pyrolysis at low temperature (300–400 °C), pyrolysis at medium temperature (400–500 °C) and pyrolysis at high temperature (>500 °C) [30,31]. The biochar produced at low and medium temperatures is hydrophobic and has more surface functional groups, but its surface area and porosity are reduced [32]. Pyrolysis at high temperatures produces biochar with increased hydrophilicity and pore structure, but the surface functional groups may be reduced [32,33].

The biochar obtained by the carbonization method performed under a temperature of 230–700 °C is considered as an effective new material, and compared with activated carbon, it presents some advantages, such as the low consumption of energy because of the low temperature used in production, the lack of requirement for a drying process, the high production yield, and the reduced ash content due to the carbonization process [24,34]. The application of biochar (BC) in environmental depollution is usually in cases of removing contaminants by adsorption [35,36]. From the consulted literature, it appears that there is a limited number of studies that refer to the use of biochars for the removal of pollutants from the air. Thus, to increase the adsorption capacity of biochars, the modification of their structure and properties is required. Generally, the modification methods of biochar refer to a treatment using various agents such as steam, CO_2_, acids, salts, and bases, which can change the biochar structure and increase their adsorption capacity [37,38].

The raw materials usually used to produce biochar are wood, agricultural waste, invasive plants, residues from the food industry, seaweed, sludge, etc. [12,22,23]. The differences in raw materials and the preparation method used can influence the physical characteristics and chemical properties, and therefore also the carbon adsorption capacity [14]. The improvement of the physical and chemical properties of the biochar is a requirement to increase its capacity to remove air pollutants. The modification methods are usually physical activation and chemical treatment. As the physical activation surface area increases, the porosity and surface functionalized groups are also increased [22,23]. The chemical modification is based on a treatment with acid or alkali agents. This method favors increasing the number of micropores, improving the biochar adsorption capacity [26,39]. Physical activation is based on a simple and low-cost method, while chemical activation usually increases micropore structure, surface area and the number of surface functionalized groups, but it is a more expensive method. Consequently, the relationship between cost and performance must be balanced when choosing the activation method.

In this work, biochars prepared by the carbonization of rapeseed cake and walnut shells were modified by treatment with H_2_SO_4_ and KOH, activated biochars (ABC) were created, and then they were tested for VOC adsorption. A polar and nonpolar compound—acetone and toluene, respectively—were used as the VOC template. The aim of this work was to evaluate the performance of this activated biochar as an effective VOC sorbent, to analyze the effects of H_2_SO_4_ and KOH activation on the VOC adsorption capacity of activated biochars and to show the VOC adsorption mechanisms of activated biochar.

## 2. Materials and Methods

### 2.1. Materials

**Figure 1:**
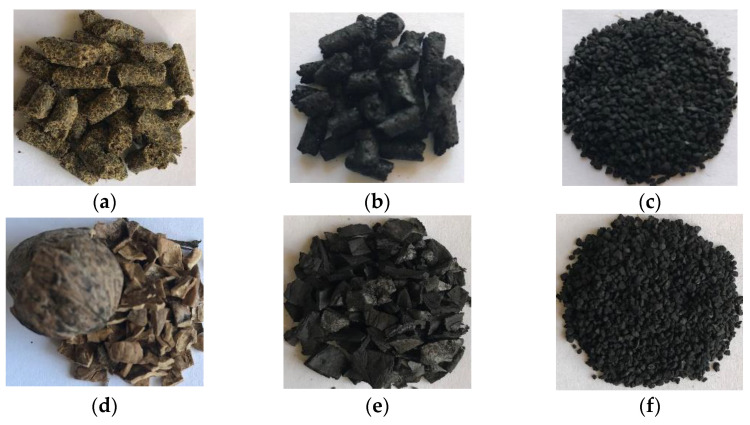
Raw biomass samples: (**a**)—rapeseed cake; and (**d**)—walnut shells; Biochars: (**b**)—rapeseed cake biochar (RSC); (**c**)—rapeseed cake biochar (RSC) with particles size ≤ 1 mm; (**e**)—walnut shell biochar (WSC); (**f**)—walnut shell biochar (WSC) with particle size ≤ 1 mm.

Renewable raw materials with high carbon content were employed as precursor materials. Rapeseed cake (RSC) and walnut shells (WS) were used to prepare biochars and activated biochars. Figure 1 shows the two kinds of raw materials that were used for producing biochars. The RSC and WS are structurally different from each other, and as a result, both raw materials produce biochar with different surface area, surface morphology, porosity and pore size.

The elemental compositions of RSC and WSC are presented in Table 1. The carbon content of raw biomass (RC and WS) were 45.91% and 47.67%, respectively.

### 2.2. Raw Material Carbonization

The main purpose of carbonization is to remove the volatile matter from the biomass in order to convert it to a suitable biochar for activation. During the phase of carbonization, the carbon content in the biochar arrives at a higher value than raw materials. Most of the non-carbon elements, and also hydrogen and oxygen, are removed in gaseous form.

Before carbonization, the rapeseed cake was in the form of extrudates (diameter of 5 mm and length of 6–8 mm) as a result of extracting the oil from the seeds (Figure 1a), while walnut shells were crushed into a particle size of ≤5 mm (Figure 1b) in order to perform homogeneous drying and carbonization. Before carbonization, both types of samples were dried for 24 h at 105 °C to remove moisture content from their mass. The carbonization for both biomass samples was performed at 650 °C at a heating rate of 5 °C/min with N_2_ flow rate of 80 mL/min for 60 min, after the set temperature was reached. The carbonization reactor was made of stainless steel, was heated electrically and was provided with a temperature controller, and a rotameter was used to control the nitrogen flow. 

The resulting biochars were rinsed for 15 min by submersion in DI water to remove the soluble matter in water and then were dried in a oven for 24 h at 80 °C. Afterwards, the biochar samples were crushed and sieved to a size of 100 μm (1 mm) for the VOC adsorption tests. The activated biochar samples were obtained by the following procedure. The biochar sample (with a particle size of 100 μm) was mixed in a 1:2 *w*/*w* ratio with either 5 M (KOH) or 5 M (H_2_SO_4_) solution. Each mixture was magnetically stirred on a hot plate for 4 h at 85 °C, and the resulting slurry was dried in a oven for 24 h at 105 °C. The dried biochar slurry was then activated in a furnace for 1 h at 800 °C under N_2_ gas flow at a rate of 100 mL/min. Afterwards, the samples were cooled to room temperature and washed repeatedly with DI water to remove any residual chemical substances and until the pH of the rinsing water was stabilized. Biochar derived from rapeseed cake and walnut shells were labeled as RSC and WSC. Those modified by KOH and H_2_SO_4_ were labeled as RSC_K_, RSC_S_, WSC_K_ and WSC_S_, respectively. The physical and chemical properties of the biochars have been determined and are shown in Table 1. 

### 2.3. Physiochemical Analysis of Biochar Samples

The porous characteristics of all samples were determined using N_2_ adsorption/desorption experiments at (−196.15 °C) using a Quantachrome Inst. (Boynton Beach, FL, USA), Nova 2200 e analyzer; the Brunauer–Emmett–Teller (BET) method was used to calculate the surface area; the pore volume distribution was at a relative pressure of P/P_o_ = 0.99 and the pore size distribution resulted from the Brunauer–Joyner–Halenda (BJH) model. The ultimate analysis determined the carbon, hydrogen, nitrogen and sulfur content, while the oxygen content was determined by difference. A Carlo Elba 1106 instrument (Elemental Microanalysis Ltd., Devon, UK) and ASTM 128 D 5373 standard were used. The ash content was determined according to the standard ASTM D3174 89. Acetone and toluene, analytical grade, provided by Sigma-Aldrich (St. Louis, MO, USA), were used as VOC templates. The VOCs’ (toluene and acetone) properties are presented in Table 2. Scanning electron microscopy (SEM) analysis was performed to study the morphological structure and a Scanning Electron Microscope type Quanta 200 was used, operating at 20 kV with secondary electrons. Additionally, FTIR analysis was performed and an FTIR spectrometer Nicolet 6700 (International Equipment Trading Ltd., Mundelein, IL, USA) was used.

### 2.4. Adsorption and Desorption of VOCs

The adsorption/desorption experiments were performed by the gravimetric method using a TGA (TGA/DSC System, Mettler Toledo, Columbus, OH, USA). Around 15 mg biochar sample was introduced into the vessel and it was degassed at 105 °C for 2 h, under a N_2_ flow of 50 mL/min. Afterwards, the sample was cooled to the adsorption temperature (room temperature, about 20 °C) and the inlet line was switched from N_2_ gas to VOCs vapor stream (50 mL/min) until the mass change arrived at an equilibrium. The toluene and acetone adsorption performance of biochars were carried out for each separately. The simulated gas consisted of 1000 ppm toluene or acetone in N_2_ used as carrier gas and the running adsorption was made at the same time (15 min). The desorption process was carried out by the increase of the temperature from room temperature to 150 °C at a heating rate of 10 °C/min. Three adsorption tests at the same conditions were performed for each experiment and an average value was considered. The data standard deviations were determined and the relative error was less than 1.5%.

## 3. Results and Discussion

### 3.1. Biochars and Chemically Activated Biochars

The feedstock type of biochar influences its physio-chemical characteristics that further determine its adsorption capacity. As shown in Figure 1, the raw biomass used in this study was: (a)—rapeseed cake and (b)—walnut shells. After the carbonization step, the samples became (c)—rapeseed cake biochar (RSC) and (d)—walnut shell biochar (WSC), which were further ground and sieved, resulting in the samples: (e)—rapeseed cake biochar (RSC) with a particle size of ≤1 mm; (f)—walnut shell biochar (WSC) with a particle size of ≤1 mm. The characteristics of biochars were affected by the activation agent and treatment conditions (Table 1). The biochar derived from walnut shells presented a higher surface area and pore volume than rapeseed cake biochar, whereas it resulted in the opposite effect after activation by KOH or H_2_SO_4_. 

Non-activated biochars (as resulting from the carbonization process) exposed low surface areas (less than 10 m^2^/g), whereas after chemical treatment with KOH or H_2_SO_4_ and thermal treatment at 800 °C, the surface area increased. In addition, the activation by H_2_SO_4_ produced higher surface area on the biochar as is shown in Table 1. Additionally, for the pore volume, a similar change was observed; the RSC_S_ presented a surface area of 1114 m^2^/g and a pore volume of 1.47 cm^3^/g, while the WSC_S_ had a smaller surface area (1020 m^2^/g) and pore volume (1.29 cm^3^/g). The difference between the surface area and the pore volume may be due to the fact that RSC contains a larger amount of volatile organic compounds [42], which can react with chemical agents and are removed from char mass, and as a consequence create larger porosity and implicitly a larger surface area (Table 1). As reported in the literature [43,44], the biochar treated with an acid and activated by thermal treatment mainly presents a microporous structure, while the biochar treated with a basic agent presents a more mesoporous structure. The different structure of the pores in the activated biochar mass is due to the different activation mechanisms of the acidic and basic agents used. The acid agent produces bond-breaking reactions, which favors the pyrolytic decomposition of the biochar and leads to the loss of the content of non-carbonic substances in the biochar mass and to the subsequent formation of micropores through carbonization [43]. Regarding the basic agent introduced into the biochar mass, it can react at high temperatures with the carbon formed of carbonates and alkaline oxides, which can further interact with the carbon and widen the structure, which therefore leads to the formation of mesopores [44]. The results obtained by us agree with these findings.

The ash content in the RSC untreated biochar was initially higher (6.24%), while after treatment and activation by H_2_SO_4_ or KOH, the ash content decreased for the RSC_S_ and RCS_K_ samples (4.16% and 4.45%, respectively). On the other hand, for the WSC_S_ and WSC_K_ samples, the ash content remained almost unchanged (Table 1). This behavior can probably be attributed to the fact that the chemical agents solubilize and remove more compounds from the initial ash in the case of the biochar prepared from rapeseed cake, while for the biochar obtained from walnut shells this process does not take place.

Regarding the oxygen content, it can be seen from Table 1 that the RSCS sample treated and activated with H_2_SO_4_ has a lower oxygen content than that contained in the untreated sample (RSC) or treated with KOH (RSC_K_). The WSC samples treated with H_2_SO_4_ or KOH (WSC_S_ or WSC_K_) also have a lower oxygen content than the untreated sample (WSC). This behavior can be explained by the fact that, in the case of biochars obtained from rapeseed cake and walnut shells and activated by H_2_SO_4_ or KOH, this probably resulted in more groups without oxygen content on the surface, while for non-treated biochars these groups were increased [45,46,47].

### 3.2. Morphological Structure of Non and Activated Biochars

The pore structure of the prepared biochars depends on the precursor biomass materials and activation methods, and represents the reason why surface area or pore volume of biochar vary from one kind to another. The porous structures of all prepared biochars were investigated using a scanning electron microscope (SEM) to study the changes in morphology during raw material carbonization and activation process.

**Figure 2:**
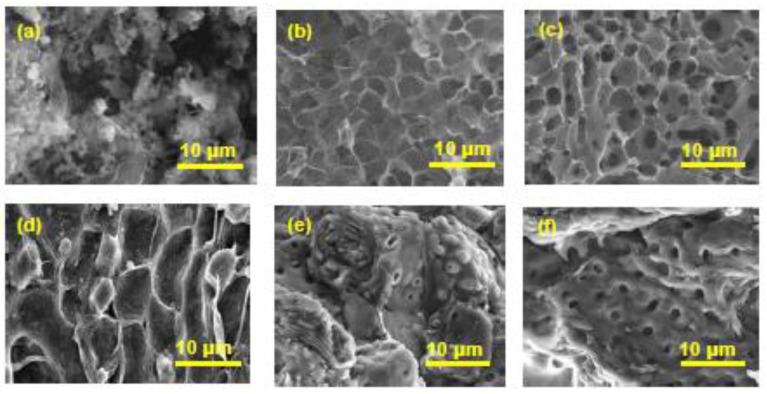
SEM images: (**a**) rapeseed cake biochar (RSC); (**b**) rapeseed cake biochar activated with KOH (RSC_K_); (**c**) rapeseed cake biochar activated with H_2_SO_4_ (RSC_S_); (**d**) walnut shell biochar (WSC); (**e**) walnut shell biochar activated with KOH (WSC_K_); (**f**) walnut shell biochar activated with H_2_SO_4_ (WSC_S_).

The SEM images of RSC and WSC materials are shown in Figure 2a,d.

The RSC biochar (Figure 2a) is composed of irregularly shaped particles with different sizes, which shows the tendency of agglomeration. The external surface of the RSC biochar presents deep slits, which could have appeared due to the removal of volatile organic compounds during the carbonization process. During the carbonization process, most of the non-carbon elements are removed, which results in a biochar retaining the shape of the biomass precursor and forming the initial porosity of the biochar.

As can be seen in Figure 2b,c, the surface area of RSC activated with KOH and H_2_SO_4_ was fairly smooth with some occasional cracks, mainly for the RSC_K_ sample. Both activated biochar samples (RSC_K_ and RSC_S_) presented well-developed and orderly pores, which correspond mainly to micropores. The use of KOH and H_2_SO_4_ in the chemical activation process led to organized surfaces on the surface of the crystalline carbon characterized by microporosity, indicating a higher surface area for the biochar sample activated with H_2_SO_4_ (Table 1). Therefore, the H_2_SO_4_ is a more effective activating agent than KOH, and activated biochar also resulted in a higher surface area. This observation is supported by the BET surface area (Table 1).

**Figure 3:**
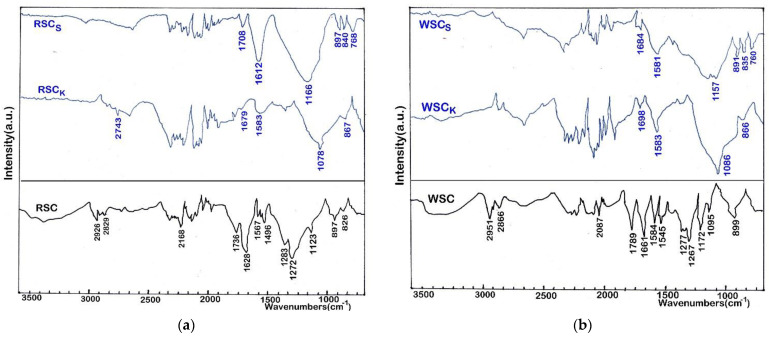
FTIR spectra of biochar: (**a**) biochar and activated biochars prepared from rapeseed cake; (**b**) biochar and activated biochars prepared from walnut shells.

As shown in Figure 2d, only a small amount of pores and low porosity existed on the surface of carbonized raw material (walnut shells). Figure 2e,f show the biochar prepared by activation using KOH and H_2_SO_4_, whose pore structure became developed. The main reasons for the pore formation are the volatilization of KOH and H_2_SO_4_ during the thermal treatment, the reaction of chemical agents with surface carbon, and the removal of some carbon particles by acid and base washing. The H_2_SO_4_ had a more significant effect on the surface morphology of biochar, showing a larger number and a more uniform pore size structure. Activation with base and acid agents effectively removed the layer on the surface of walnut shells biochar, allowing the internal structure to be revealed (Figure 2e,f). Additionally, a higher surface area resulted for the biochar sample activated with H_2_SO_4_ (Table 1). It was reported that the type and number of chemical functional groups on the surface adsorbent material has a great influence on adsorption capacity and selectivity. In order to analyze the surface chemical functional group of biochar samples, FTIR analysis was performed and an FTIR spectrometer Nicolet 6700 was used. The dried sample was blended with KBr and the resulting mixture was placed in the sample holder. The FTIR spectra are presented in Figure 3a,b.

As can be seen, in both cases there is a clear difference between the spectra of the untreated biochars and the biochars treated with KOH and H_2_SO_4_. For untreated biochars the peaks at 2926 cm^−1^ and 2829 cm^−1^ for RSC and 2951 cm^−1^ and 2866 cm^−1^ for WSC, respectively, are associated with alkyl groups, which can be correlated with the hydrophobic nature of biochars. The peaks around 1736 cm^−1^ for RSC and 1789 cm^−1^ for WSC, respectively, correspond to the C=O (carboxyl) stretch, while the peak around 1628 cm^−1^ for RSC and 1661 cm^−1^ for WSC, respectively, are associated with the C=C stretch (aromatics). The peaks at 1567 cm^−1^ for RSC and 1584 cm^−1^ for WSC, respectively, are associated with C–H or N–H bonds. On the other hand, the peaks at 1123 cm^−1^ for RSC and 1095 cm^−1^ for WSC, respectively, are associated with the C–O group from esters and ethers. The FTIR spectra of the treated biochars (RSC_K_, RSC_S_, WSC_K_ si WSC_S_) showed that chemical activation using H_2_SO_4_ produced biochars with a much more active surface, as shown by the number of peaks and intensity. The biochars treated with H_2_SO_4_ have strong peaks at 1612 to 1581 cm^−1^ (aromatic C=C stretching or conjugated C-O stretching) and ~1166–1157 cm^−1^ (C–O–C stretching of ester groups). The biochars treated with KOH also have a peak around the same region, but of much lower intensity than with the biochars treated with H_2_SO_4_. (see Figure 3a,b). The biochars treated with KOH presented a strong peak at ~1078 cm^−1^ for RSC_K_ or at ~1086 cm^−1^ for WSC_K_, respectively, corresponding to the C-O stretching of carboxylic, ester and ether groups. The disappearance of peaks in the range 1078 to1086 cm^−1^ for the biochars treated with H_2_SO_4_ is probably due to dehydration and depolymerization reactions during the activation [43,48].

### 3.3. VOCs Removal

The VOC compounds can be sorted into the following categories: alkanes, alkenes, aromatic hydrocarbons, halogenated hydrocarbons, aldehydes, ethers, esters, ketones, organic acids, and others. Most VOC compounds are released during the use of fossil fuels, and they are very important precursors to the formation of other secondary pollutants and fine particulate matter. These compounds can cause air pollution by smog forming [47]. The removal of VOCs using activated biochars has been the subject of much research [8,14,22]. For example, Zhang et al. [14] prepared biochars using hickory wood and peanut hull and used them to adsorb acetone and cyclohexane. After treatment with H_3_PO_4_, the surface area of the biochars increased by 155 and 180 times, respectively, and the biochar obtained from hickory wood presented a large capacity for acetone removal. Kinetic analysis showed that the physical adsorption played a key role in the VOC removal [48]. Lamplugh et al. [49] used a biochar to remove acetone, and it was observed that the adsorption capacity was influenced by the porosity, surface area and average pore size of the biochar. Regarding the mechanisms of removal, it was shown that physical adsorption was the prevailing mechanism for VOC removal using biochar. The adsorption data can be described by the models pseudo-first-order, according to Equation (1), and pseudo-second-order, according to Equation (2):

q_t_ = q_e_ (1 × 10^−k1t^) (1)

q_t_ = t·k_2_·q_e_^2^/1 + q_e_·t·k_2_ (2)

where q_t_ (mg/g) and q_e_ (mg/g) are the amount of VOCs adsorbed at time t (min) and equilibrium, respectively; k_1_ (min^−1^) and k_2_ (g/mg·min) are adsorption rate constants.

The pseudo-second-order model fit the VOC adsorption on carbonaceous adsorbents better than the pseudo-first-order model, which was consistent with the other literature [50,51,52]. According to this data, the pseudo-second-order model corresponds better to the adsorption of VOCs on carbonic adsorbents than the first-order model. In these studies, it is emphasized that the specific surface area of carbonic adsorbents is mainly responsible for the physical adsorption of organic compounds. Therefore, biochars with a larger surface area may have higher adsorption rate constants, implying that physical adsorption may be the dominant mechanism for biochars with a larger surface area. As can be seen in Table 1 and Figure 3, RSC_S_ presented the highest specific surface (1114 m^2^/g) and had a higher adsorption capacity for acetone and toluene. Additionally, as presented in the literature [53,54], VOC adsorption involves two parts: VOC adsorption at the surface and diffusion in the pores. Moreover, intraparticle diffusion plays a major role, which could affect the adsorption rate [54].

Other investigations showed that π–π interaction can be an important removal mechanism for VOCs when biochar is used, especially for benzene removal [14]. Due to the various different characteristics of VOCs, the mechanisms of VOC removal using activated and non-activated biochars still requires further investigation. It is necessary to use suitable raw materials and preparation methods to improve the biochar properties that are important for the removal of a variety of VOC pollutants. The activated biochars prepared in this study were evaluated in terms of their performance in the adsorption of toluene and acetone, compounds that are part of VOCs. 

### 3.4. VOC Adsorption on Biochar Samples

**Figure 4:**
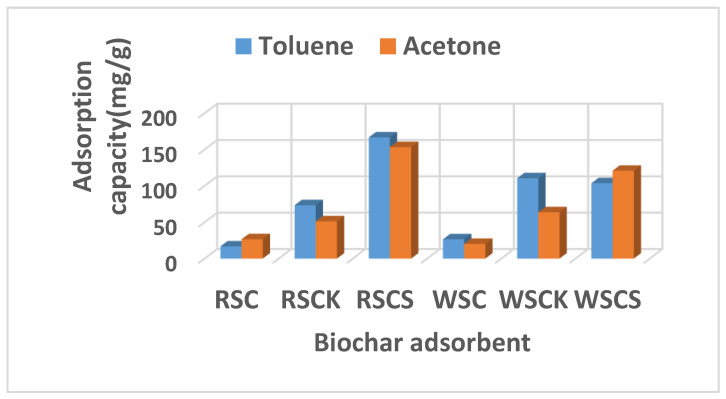
The adsorption capacity of toluene and acetone (VOCs) on different activated and non-activated biochar samples at room temperature (20 °C).

Figure 4 presents the adsorption capacity of toluene and acetone (VOC templates) on different activated and non-activated biochar samples prepared in this work at room temperature (20 °C).

The adsorption capacity of toluene and acetone on RSC and WSC biochars were low, as can be seen in Figure 4, the values being in the range of 16.76–26.65 mg/g, whereas after activation by KOH the adsorption capacity of biochars increased significantly to 51.28–73.46 mg/g for RSC_K_ and to 63.72–110.53 mg/g for WSC_K_, respectively. Activation with H_2_SO_4_ increased the adsorption capacity to 153.34–166.72 mg/g for RSC_S_ and at 103.68–120.85 mg/g for WSC_S_, respectively (Figure 4).

Generally, most of the H_2_SO_4_-activated biochars have a higher adsorption capacity than that of KOH-activated biochars. The toluene and acetone adsorption capacity on RSC_S_ was 153.34 mg/g and 148.25 mg/g and on WSCS was 98.77 mg/g and 120.85 mg/g, respectively which are bigger than that on RSC_K_ and WSC_K_. A greater amount of acetone was adsorbed on H_2_SO_4_-activated biochars than that on KOH-activated biochars, which may be due to the following aspects: firstly, the biochar activated with H_2_SO_4_ has a larger surface area, which means that it can offer more adsorption sites for sorbate molecules (acetone, toluene) and the second aspect refers to the fact that the activation with the acid agent (H_2_SO_4_) possibly generated functional groups containing oxygen on the surface of the biochar, which can improve the adhesion of the hydrophilic VOC compound (acetone) on the surface of the biochar.

A similar behavior of activated carbons obtained by treatment with an acid was also presented in other studies [55,56]. These adsorbents presented a higher adsorption capacity to polar VOC compounds, but at the same time it was found that the presence of groups containing oxygen on the carbon surface decreased the adsorption capacity for nonpolar VOC compounds, determining the inhibition of the interactions between nonpolar VOCs and the adsorption sites rich in p-electrons. However, as shown in other studies [46,57], activation with a basic agent can remove functional groups containing oxygen from the surface of the carbonaceous material. This may be the reason why, when RSC and WSC were activated with KOH, the resulting activated biochars (RSC_K_ and WSC_K_) presented a higher toluene (nonpolar VOC) adsorption capacity (73.46 and 103.68 mg/g, respectively), although the surface area of the RSC_K_ and WSC_K_ was almost 2.5 and 5 times lower than biochars activated with H_2_SO_4_ (Table 1). 

These data are in agreement with results presented in other studies [46,57,58], such as lower adsorption capacities for activated carbons with an acid being obtained for nonpolar VOCs (o-xylen, benzene, or CCl_4_). The toluene adsorption capacity of most of the used activated biochars was bigger than that of adsorption capacity corresponding to acetone. This can be correlated with the characteristics of VOC molecules, such as molecular weight, boiling point, density, etc. (as shown in Table 2).

The VOCs’ molecular weight can influence their adsorption on an adsorbent, and this was confirmed in the study from reference [59]. In this study, the adsorption of VOCs on sludge-derived carbonaceous materials was compared, showing that the diffusion coefficients of bigger molecular weight VOCs were lower than that of VOCs with higher molecular weight. In our study, toluene has a higher molecular weight (92.141 g/mol) than acetone (58.08 g/mol); however, it had a larger adsorption capacity than acetone. This is possibly due to the non-polar character of the toluene molecule. In addition, the VOC adsorption on a porous adsorbent material can be compared to vapor–liquid phase transition (condensation), when the VOC compound with a higher boiling point is adsorbed due to their stronger interaction with the adsorbent material by intermolecular forces [34]. Therefore, for toluene, which has a higher boiling point (111 °C) than acetone (56.05 °C) (Table 2), the prepared biochars had higher adsorption capacities (Figure 4). On the other hand, it is more difficult for VOC compounds with a higher boiling point to desorb due to their stronger affinity with the surface of the adsorbent material.

**Figure 5:**
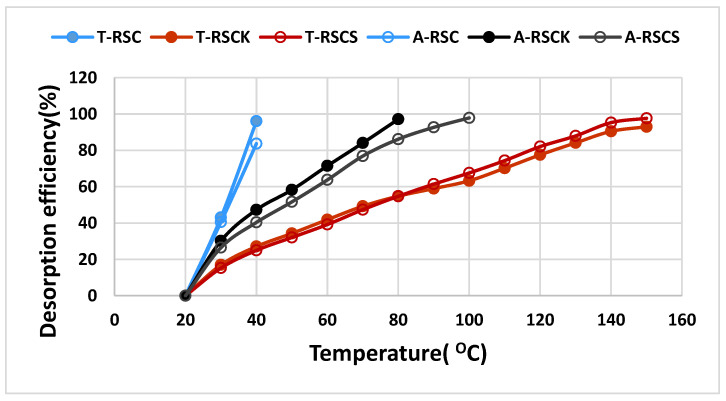
Efficiency of toluene and acetone desorption (T-RSC, T-RSC_K_, T-RSC_S_ and A-RSC, A-RSC_K_, A-RSC_S_) from nonactivated (RSC) and activated (RSC_K_, RSC_S_) rapeseed cake biochars.

One of the most important factors that contributes to the commercial use of an adsorbent material is the one that ensures reuse (lifetime). To investigate the reuse of biochars, the desorption process was carried out using TGA and samples of biochars saturated with VOCs (toluene, acetone), at a heating rate of 10 °C in the temperature range from 20 °C to 150 °C. Figure 5 shows the efficiency of toluene and acetone desorption process. 

As can be seen in Figure 5, toluene and acetone adsorbed on non-activated RSC were completely desorbed at temperatures ≤40 °C, while those on activated RSC (RSC_K_ and RSC_S_) were desorbed at higher temperatures (80–100 °C) for acetone and (140–150 °C) for toluene. The desorption temperature for toluene was higher than that of acetone; this could be due to the fact that toluene has a larger kinetic diameter (Table 2) and the release of toluene molecules from the micropores was inhibited, and the toluene adsorbed in the narrow micropores required a higher temperature for desorption.

**Figure 6:**
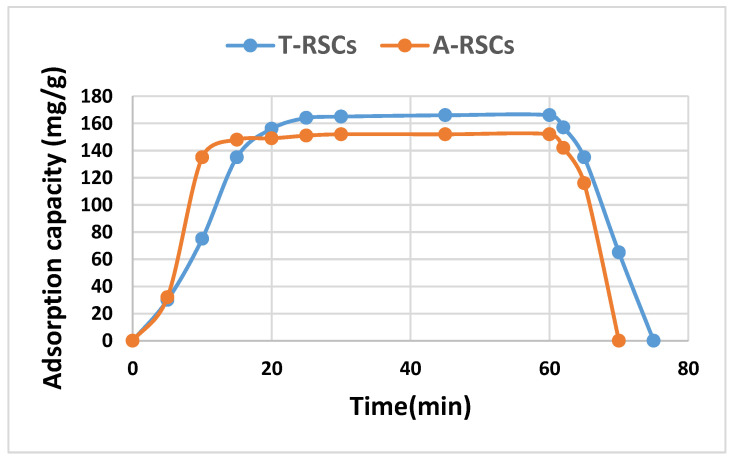
Adsorption capacity of toluene and acetone on rapeseed cake biochar activated with H_2_SO_4_ (T-RSCs and A-RSCs).

On the other hand, the highest toluene and acetone adsorption capacities were obtained on RSC_S_ (rapeseed cake biochar activated with H_2_SO_4_), Figure 4. For this biochar, the breakthrough curves are shown in Figure 6. 

Both the toluene and acetone adsorption capacities on RSC_S_ increased in the first 10–20 min, reaching a maximum value of 166 mg/g for toluene and 152 mg/g for acetone, respectively, values that remained for around 40 min, after which they decreased rapidly, which indicated the complete saturation of the material with VOCs.

Table 3 shows the adsorption capacity for biochars prepared in this work and other materials from other studies [60,61,62,63,64]. The data indicate a good adsorption capacity of activated biochars prepared in this work, these being comparable with other results presented in the literature (Table 3). 

### 3.5. Main Factors Influencing Adsorption on Activated Biochars of VOCs

Characteristics of adsorbent materials, such as specific surface area and pore structure, together with functional groups on the surface area of adsorbents, are three key factors which could directly influence their performance on VOC removal by adsorption [64,65,66,67]. Carbon materials with a large specific surface area usually have superior adsorption performance because their surface area provides the sites for adsorption of VOC molecules. Alkaline metal compounds are commonly used for biochar activation, and they can produce large surface areas and highly microporous structures. Activation with KOH has been proposed to proceed as 6KOH + 2C → 2K + 3H_2_ + 2K_2_CO_3_ [68,69], where the reactivity of the precursor has been linked to the start of hydrogen evolution during heating. When hydrogen evolution begins earlier, the precursor is more reactive towards KOH. H_2_SO_4_ is another efficient activator agent; it can be efficiently absorbed in the pores of a material and is used as an impregnation chemical in activation. The suitability of H_2_SO_4_ for activation of biochars is clear; the low surface area of RSC and WSC biochars was improved (see Table 1). This is probably due to the different activation mechanisms, as KOH reactions are oxidative, but with H_2_SO_4_ the pore structure formation is based on the dehydration reactions and the occupation of the acid in the pores, which prevents destructuring. The content of functional groups on the biochars influence the VOC adsorption. As is mentioned in the literature [70,71], the correlations between the amount of different surface chemical groups and adsorption capacities revealed differences between the adsorbent materials. As it appears from the FTIR analysis (Figure 3), the prepared biochars present phenolic, carbonyl, and carboxyl functional groups that are correlated with the hydrophilic surface of the material, while the C-H, N-H or C=C groups are correlated with the hydrophobicity of the surface. The results confirm that activation with H_2_SO_4_ and KOH produce different content of superficial surface on activated carbons, which may have strong influence on selectivity and sorption capacity of VOC applications. 

The surface area of the RSCs and WSCs samples prepared in this study (Table 1) (1114 m^2^/g and 1020 m^2^/g, respectively) is comparable to that presented in another study (Table 3), and toluene adsorption capacity is similar. A carbon material with a greater surface area captures a greater amount of VOCs. As a result, the preparation of carbonaceous materials with a large specific surface area is desirable to use in VOC capture. Pore size shows the morphology structure of a carbonaceous material, and the pore size distribution is particularly responsible for their VOC adsorption capacity [58,64]. Their pores can be classified as micropores (Dp < 2 nm), mesopores (2 nm < Dp < 50 nm), and macropores (Dp > 50 nm). As is presented in other studies [46,58,64], in general, the micropores provide main adsorption sites, while the mesopores increase the intra-particle diffusion and decrease the adsorption time. VOC adsorption onto carbon materials is influenced by pore size in various ways; micropores, particularly the narrow micropores, dominate VOC adsorption onto carbon materials [72,73]. It has been reported [74,75] that narrower micropores (size < 1 nm) contribute to a better adsorption capacity for VOCs than the total micropore volume [67]. As can be seen from Table 1, the RSCs and WSCs biochars prepared in this study present micropores with diameter < 1 nm of 0.89 nm and 0.97 nm, respectively, which confirms the good behavior of these materials in the process of VOC capturing (toluene and acetone).

The VOC adsorption of carbonaceous adsorbent materials is usually controlled by physical and chemical processes. Besides the morphology structure, the surface chemical functional groups of carbon materials also influence their adsorption of VOCs [65]. The surface functional groups of carbonaceous materials can be associated with both with the raw material and with the material activated by thermal and chemical treatment [76]. The oxygen groups are of different types such as acidic, basic, or neutral. Most of the oxygen groups represent the source of surface acidity, which favor the hydrophilic VOCs to be adsorbed on the carbon material surface [64,65]. The treatment by acids produces the surface oxidations and introduces surface oxygen groups to carbonaceous materials [77]. RSCs and WSCs resulted from treatment using H_2_SO_4_, which implies that oxygen groups appeared on the surface of these biochars, and therefore the adsorption capacity for toluene and acetone was improved, as can be seen in Figure 3. A carbon material with a high adsorption capacity for VOCs will be regenerated at longer time intervals [66], which means a lower cost for a process of removing VOCs from the air.

Another factor that influences the adsorption capacity of carbon materials is the molecular polarity of VOCs. Usually, the polar VOCs are adsorbed on adsorbent materials which have polar groups, while nonpolar VOCs are adsorbed easier on the adsorbents without polar groups. Comparing various VOC adsorptions on carbonaceous adsorbents [64,65,66,74], the results show that nonpolar VOCs exhibited higher adsorption capacity on biochar and activated carbon than other polar VOCs, and this is attributed to their zero dipole moment, which is well matched with the carbonaceous adsorbents. As can be seen in Table 2, toluene has zero dipole moments, which explains the higher value of the adsorption capacity for this than for acetone (Figure 4 and Figure 6).

In addition, the boiling point of VOCs influence their adsorption process. The physical adsorption on a porous adsorbent material is similar to vapor–liquid phase transition, where the adsorbate with a higher boiling point could be preferentially adsorbed compared to those with a lower boiling point because of the stronger intermolecular forces [78]. The same finding also emerges from the data obtained in this study; toluene, with a higher boiling point than acetone (Table 2), was adsorbed in a larger amount on prepared activated biochars (Figure 4). Because of the strong affinity between high-boiling-point VOCs and adsorbent materials, they will easily replace the lower-boiling-point VOCs during the competitive adsorption process. The adsorption performance of VOCs with a high boiling point is superior to those of VOCs with a low boiling point, whereas the desorption process of high-boiling-point VOCs is more difficult to achieve because of their strong affinity with adsorbent surfaces.

### 3.6. Effects of VOCs on Environment and Human Health, and Abatement Methods

VOCs have a negative impact on both the environment and human health. They affect human health, and most VOCs, such as aldehydes, ketones, alcohols, acids, aromatic compounds, polycyclic aromatic hydrocarbons, etc., are highly toxic and carcinogenic. Even low-level exposure to aldehydes, ketones, and alcohols can cause respiratory problems such as throat irritation, difficulty breathing, eye irritation, and chest tightness [79]. On the other hand, high concentration or long-term exposure increases the risk of acute intoxication or chronic toxicity, as well as the occurrence of nasal tumors [15,79]. Aromatic compounds, another group of VOCs, mainly including benzene, toluene and ethylbenzene, are toxic and carcinogenic [79]. Low-concentration exposure produces confusion, fatigue and nausea, as well as memory and vision loss. Exposure to higher concentrations causes unconsciousness, dizziness and even death [79]. Inhalation of benzene or toluene vapors is a major cause of leukemia; it can harm human beings specifically and systematically [80]. A concentration of approximately 2% benzene/toluene in the air can be fatal if the exposure lasts 5–10 min, because the maximum allowed exposure concentration is 16.25 μg/m^−3^ [80]. Other VOCs, such as polycyclic aromatic hydrocarbons (PAHs), including mainly naphthalene, phenanthrene and pyrene, are carcinogenic compounds [79,80]. In addition to their negative effects on health, VOCs have negative effects on the environment, contributing majorly to the effect of global warming and the depletion of the ozone layer. The threshold concentration of aromatic compounds is 200 ppm in air [79,80].

Taking into account this negative impact determined by VOCs, it is necessary to develop effective methods of removing them from the air to avoid their inhalation by people and at the same time to protect the environment from their negative effects. VOC control methods can generally be divided into recovery (capture) methods and destruction methods. The recovery methods include the adsorption process, which is based on the use of absorbent porous materials. Compared to destruction methods, which convert VOCs mainly to CO_2_ and H_2_O, recovery methods are more economical because they can achieve VOC capture and recovery. Among the recovery methods, adsorption-based technology is considered one of the most feasible methods to capture VOCs, mainly because it has low cost and high efficiency [59,64]. The use of the optimal porous solid adsorbent is crucial for the commercial application of the adsorption method for the capture of VOCs. Despite some inherent disadvantages, such as the blocking of pores present and hygroscopicity, carbon materials have great potential as adsorbents; they have low costs, good stability and high efficiency for the capture and reduction of VOCs [64,65,66]. The adsorption capacity of COVs of carbonaceous materials varies from tens of milligrams to hundreds of milligrams per gram, depending on the physico-chemical properties of the carbonaceous materials (as mentioned in Section 3.5) Based on the experimental data obtained in this study, the activated biochars prepared from residual biomass (rapeseed cake and walnut shells) could be used with good results as adsorbent materials to equip air purification filters for the capture and removal of VOCs because of their low production cost, good stability and performance.

## 4. Conclusions

Biochars derived from rapeseed cake (RSC) and walnut shells (WSC), as well as activated biochars resulting from treatment with H_2_SO_4_ and KOH, were prepared and investigated to determine their performance in terms of VOC adsorption. Their adsorption and desorption capacity for toluene (non-polar compound) and acetone (polar compound) was determined. After activation by chemical treatment with H_2_SO_4_ and KOH, the BET surface area of activated biochars increased significantly, and activation with H_2_SO_4_ created a higher surface area for biochars than that of KOH-activated biochars. The adsorption capacities of toluene and acetone for non-activated biochars were reduced (26.65 mg/g), while that for activated biochars increased quite significantly, up to 166.72 mg/g. Additionally, the biochars activated with H_2_SO_4_ presented a higher adsorption capacity than the biochars activated with KOH. The higher adsorption capacity of biochars activated with H_2_SO_4_ can be attributed to their large surface area, and also to their larger pore volume.

Based on the experimental data obtained in this study, the activated biochars prepared could be used with good results as adsorbent materials to equip air purification filters for the capture and removal of VOCs because of their low production cost, good stability and performance. Although great progress has been made in the adsorption of VOCs on carbonaceous materials, there are still gaps in knowledge that need to be filled. Further studies are still needed to further improve the VOC absorption capacity of carbonaceous materials, increase the adsorption performance of low-boiling VOCs, decrease the desorption temperature of high-boiling VOCs, increase the selectivity of carbonaceous adsorbents for the reuse of VOCs and also improve the adsorption of VOCs on carbonic adsorbents in the presence of moisture. Future research will address the development of low-cost adsorbents, which can efficiently retain pollutants from the air and thus reduce the risk to the environment and human health. In this context, activated biochars will be considered a suitable material for such applications. Biochars with a high porous structure may be obtained by the pyrolysis process of other different kinds of biomass and the parameters process may be optimized. The differences that exist between the raw materials used, as well as between the preparation methods, will influence the physico-chemical properties and implicitly the adsorption capacity of the biochar. Due to its properties, such as low cost and high adsorption capacity, the activity of functional groups on the surface of biochars could be used in systems for removing air pollutants, such as VOCs.

## Figures and Tables

**Table 1:** The main characteristics of biochars and activated biochars prepared by carbonization at 650 °C from rapeseed cake and walnut shells and activated by KOH and H_2_SO_4_ at 800 °C.

Sample	SA_BET_	P_V_	Dp	C	H	N	S	O	Ash	O/C
	(m^2^/g)	(cm^3^/g)	(nm)	(wt.%)	(wt.%)	(wt.%)	(wt.%)	(wt.%)	(wt.%)	(mol/mol)
RSC	8.5	0.124	0.92	46.05	6.14	6.68	0.85	39.62	6.24	0.645
WSC	9.6	0.126	1.12	47.62	5.72	0.35	0.68	45.63	7.26	0.718
RSC_K_	583	0.64	67.56	85.52	1.85	1.85	0.63	10.14	4.45	0.089
RSC_S_	1114	1.47	89.16	91.71	1.73	1.28	0.62	4.65	4.05	0.038
WSC_K_	235	0.47	57.45	83.34	1.76	0.32	0.64	13.93	6.45	0.125
WSC_S_	1020	1.29	97.05	89.73	1.87	0.28	0.37	7.74	6.12	0.064

SA_BET_—surface area; P_V_—pore volume; Dp—pore diameter.

**Table 2:** Some characteristics of toluene and acetone used as VOC templates in adsorption experiments [40,41].

VOCs	F_M_	W_M_ (g/mol)	ρ (g/mL)	Bp (°C)	Dm (D)	D_K_ (nm)
Toluene	C_7_H_8_	92.141	0.87	111	0	0.58
Acetone	C_3_H_6_O	58.08	0.7845	56.05	2.91	0.46

F_M_—Molecular formula; W_M_—Molecular weight (g/mol); ρ—Density (g/mL); Bp—Boiling point (°C); Dm—Dipol moment (D); D_K_—Kinetic diameter (nm).

**Table 3:** The adsorption capacity of the prepared biochars compared to data from the literature.

Material	S_BET_	Adsorption Capacity (mg/g)	Reference
Commercial AC	934	41	[60]
AC (rice husk)	1818	264	[61]
AC/ZrO	837	127	[60]
AC (petroleum waste)	2692	659.9	[62]
AC-Z	795	258	[63]
HPC-900	578	182	[64]
RSC_S_	1114	166.72	[This work]
WSC_S_	1020	152.34	[This work]

## Data Availability

Not applicable.
